# *catena*-Poly[iron(II)-bis­{μ-5-carb­oxy-2-[(1*H*-1,2,4-triazol-1-yl)meth­yl]-1*H*-imidazole-4-carboxyl­ato}]

**DOI:** 10.1107/S1600536811036026

**Published:** 2011-09-30

**Authors:** Yan Tong, Hui-Jie Wang

**Affiliations:** a Department of Quality Examination and Management, Zhengzhou College of Animal Husbandry Engineering, Zhengzhou, Henan, 450011, People’s Republic of China; b Department of Biological Engineering, Zhengzhou College of Animal Husbandry Engineering, Zhengzhou, Henan, 450011, People’s Republic of China

## Abstract

In the title coordination polymer, [Fe(C_8_H_6_N_5_O_4_)_2_]_*n*_ {or [Fe*L*_2_]_*n*_,where H*L* = 2-[(1*H*-1,2,4-triazol-1-yl) meth­yl]-1*H*-imidazole-4,5-dicarb­oxy­lic acid)}, the Fe^II^ ion, located on an inversion centre, is six-coordinated by two O atoms and four N atoms from two *L* ^−^ ligands in a distorted octa­hedral geometry [Fe—O = 2.1452 (13), Fe—N = 2.1316 (14) and 2.2484 (15) Å]. There is an intra­molecular O—H⋯O hydrogen bond in each *L* ^−^ ligand. Being an effective tridentate bridging ligand, the deprotonated *L* ^−^ anions link two Fe^II^ atoms, yielding a chain-like polymer propagating along [100]. In the crystal, these polymer chains are linked *via* N—H⋯N hydrogen bonds, forming a two-dimensional network.

## Related literature

For the design and self-assembly of metal-organic coordination polymers (MOCP’s), see: Batten & Robson (1998 ▶); Eddaoudi *et al.* (2001 ▶). For related structures, see: Wang *et al.* (2008 ▶); Meng *et al.* (2009 ▶); Zhang, Li *et al.* (2010 ▶); Zhang, Ma *et al.* (2010 ▶); Feng *et al.* 2010 ▶); Li *et al.* (2010 ▶); Chen *et al.* (2010 ▶); Jing *et al.* (2010 ▶). 
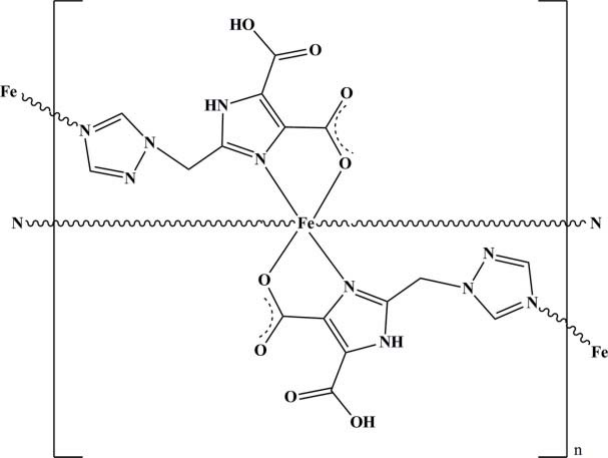


## Experimental

### 

#### Crystal data

[Fe(C_8_H_6_N_5_O_4_)_2_]
*M* *_r_* = 528.21
Monoclinic, 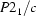

*a* = 7.1790 (14) Å
*b* = 13.490 (3) Å
*c* = 10.129 (2) Å
β = 99.11 (3)°
*V* = 968.6 (3) Å^3^
*Z* = 2
Mo *K*α radiation
μ = 0.85 mm^−1^
*T* = 293 K
0.30 × 0.15 × 0.10 mm

#### Data collection

Rigaku Mercury CCD diffractometer
Absorption correction: multi-scan (*CrystalClear*; Rigaku, 2000 ▶) *T*_min_ = 0.784, *T*_max_ = 0.919
10179 measured reflections
1900 independent reflections
1847 reflections with *I* > 2σ(*I*)
*R*_int_ = 0.020

#### Refinement

*R*[*F* ^2^ > 2σ(*F* ^2^)] = 0.027
*wR*(*F* ^2^) = 0.073
*S* = 1.08
1900 reflections
160 parameters
H-atom parameters constrained
Δρ_max_ = 0.25 e Å^−3^
Δρ_min_ = −0.25 e Å^−3^

### 

Data collection: *CrystalClear* (Rigaku, 2000 ▶); cell refinement: *CrystalClear*; data reduction: *CrystalClear*; program(s) used to solve structure: *SHELXS97* (Sheldrick, 2008 ▶); program(s) used to refine structure: *SHELXL97* (Sheldrick, 2008 ▶); molecular graphics: *SHELXTL* (Sheldrick, 2008 ▶); software used to prepare material for publication: *SHELXTL*.

## Supplementary Material

Crystal structure: contains datablock(s) global, I. DOI: 10.1107/S1600536811036026/su2292sup1.cif

Structure factors: contains datablock(s) I. DOI: 10.1107/S1600536811036026/su2292Isup2.hkl

Additional supplementary materials:  crystallographic information; 3D view; checkCIF report

## Figures and Tables

**Table 1:** Hydrogen-bond geometry (Å, °)

*D*—H⋯*A*	*D*—H	H⋯*A*	*D*⋯*A*	*D*—H⋯*A*
O3—H3*A*⋯O2	0.82	1.70	2.5175 (19)	179
N5—H5*A*⋯N2^i^	0.86	2.01	2.850 (2)	166

Symmetry code: (i) 
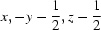
.

**Fig. 1.**
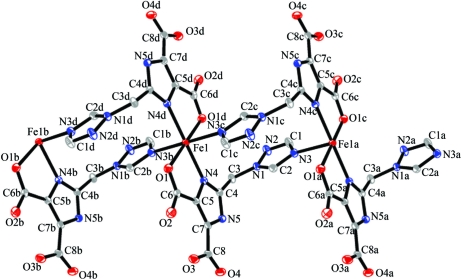
A view of the molecular structure of the title coordination polymer, showing 30% probability displacement ellipsoids and the atom-numbering [symmetry codes: (a) = -1+x, y, z; (b) = 1+x, y, z; (c) = -1-x, -y, -z; (d) = -x, -y, -z].

**Fig. 2.**
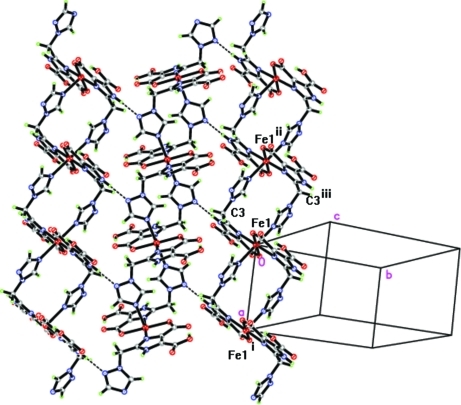
A view perpendicular to the two-dimensional network structure of the title polymer, formed via N-H···N hydrogen bonds (dashed lines; see Table 1 for details).

## supplementary crystallographic information

### Comment

The design and self-assembly of metal-organic coordination polymers (MOCP's) has received much attention since the early work of (Batten & Robson, 1998) and the group of Yaghi (Eddaoudi *et al.*, 2001). The selection of suitable bi- or multi-dentate bridging ligands plays a crucial role in the construction of MOCP's, through tuning their structural dimensionalities and stereochemistry with different coordination sites. In this work, the semi-rigid ligand, 2-[(1*H*-1,2,4-triazol-1-yl) methyl]-1*H*-imidazole-4,5-dicarboxylic acid (HL), was synthesized. Similar ligands, which differ only in the groups on the 2-position of the imidazole ring, such as 2-ethyl-1*H*-imidazole-4,5-dicarboxylic acid, 2-propyl-1*H*-imidazole-4,5-dicarboxylic acid (Wang *et al.*, 2008; Meng *et al.*, 2009; Zhang, Li *et al.*, 2010; Zhang, Ma *et al.*, 2010; Feng, *et al.*, 2010), and 2-pyridinyl-1*H*-imidazole -4,5-dicarboxylic acid (Li *et al.*, 2010; Chen *et al.*, 2010; Jing *et al.*, 2010), have been studied extensively. We report herein on the crystal structure of the title one-dimensional coordination polymer.

As shown in Fig. 1, the asymmetric unit of the title coordination polymer contains half a Fe^II^ ion, located on an inversion centre, and a deprotonated *L*^-^ ligand. The local coordination geometry around the Fe^II^ centre can be described as distorted octahedral. The equatorial plane is formed by two imidazole N atoms (N4 and N4d) and two carboxylate O atoms (O1 and O1d) from two L^-^ ligands, while the axial positions are occupied by two triazolate N atoms (N3b and N3c). The *cis* bond angles around each Fe^II^ centre are in the range 78.11 (5)° to 101.89 (5)°.

Two Fe^II^ centers are linked together by two identical *L*^-^ ligands through triazolate N-donors, imidazole N-donors and carboxylate O-donors into a 14-membered box-like macrocycle with the Fe1···Fe1^ii^separation being 7.179 Å. The symmetrically related triazolyl rings are parallel to one another, and the shortest distance between atoms is 3.662 Å, indicating a weak π-π interaction. Being an effective tridentate bridging ligand, the deprotonated *L*^-^ anions link two Fe^II^ centers to yield a one-dimensional chain-like polymer propagating along [100].

In the crystal the one-dimensional polymer chains are linked by classical N-H···N hydrogen bonds, involving the uncoordinated triazolate N atoms and the imidazole N atoms, resulting in the formation of a two-dimensional supramolecular network propagating in the *bc*-plane (Table 1 and Fig. 2).

### Experimental

The title coordination polymer was synthesized by adding 1.0 mmol of 2-[(1*H*-1,2,4-triazol-1-yl) methyl]-1*H*-imidazole-4,5-dicarboxylic acid, (HL), to 5 mL water. Then FeSO_4_(0.5 mmol) was added to the above solution, and the mixture was heated to 393 K for 3 days, and then cooled to room temperature. Yellow crystals, suitable for X-ray analysis, were obtained in 46% yield. Anal. Calcd (%) for C_16_H_12_FeN_10_O_8_: C, 36.38; H, 2.29; N, 26.52. Found (%): C, 36.52; H, 2.45; N, 26.36.

### Refinement

The H atoms were included in calculated positions and treated as riding atoms: C–H = 0.93 Å for the triazole and 0.97 Å for the methylene H atoms with *U*_iso_(H) = 1.2*U*_eq_(C); O–H = 0.82 Å and N–H = 0.86 Å, with *U*_iso_(H) = 1.5*U*_eq_(O) and 1.2*U*_eq_(N).

### Crystal data

**Table:**

[Fe(C_8_H_6_N_5_O_4_)_2_]	*F*(000) = 536
*M**_r_* = 528.21	*D*_x_ = 1.811 Mg m^−^^3^
Monoclinic, *P*2_1_/*c*	Mo *K*α radiation, λ = 0.71073 Å
Hall symbol: -P 2ybc	Cell parameters from 3321 reflections
*a* = 7.1790 (14) Å	θ = 2.0–31.1°
*b* = 13.490 (3) Å	µ = 0.85 mm^−^^1^
*c* = 10.129 (2) Å	*T* = 293 K
β = 99.11 (3)°	Prism, yellow
*V* = 968.6 (3) Å^3^	0.30 × 0.15 × 0.10 mm
*Z* = 2	

### Data collection

**Table:**

Rigaku Mercury CCD diffractometer	1900 independent reflections
Radiation source: fine-focus sealed tube	1847 reflections with *I* > 2σ(*I*)
graphite	*R*_int_ = 0.020
ω scans	θ_max_ = 26.0°, θ_min_ = 2.5°
Absorption correction: multi-scan (*CrystalClear*; Rigaku, 2000)	*h* = −8→8
*T*_min_ = 0.784, *T*_max_ = 0.919	*k* = −16→16
10179 measured reflections	*l* = −12→12

### Refinement

**Table:**

Refinement on *F*^2^	Primary atom site location: structure-invariant direct methods
Least-squares matrix: full	Secondary atom site location: difference Fourier map
*R*[*F*^2^ > 2σ(*F*^2^)] = 0.027	Hydrogen site location: inferred from neighbouring sites
*wR*(*F*^2^) = 0.073	H-atom parameters constrained
*S* = 1.08	*w* = 1/[σ^2^(*F*_o_^2^) + (0.0398*P*)^2^ + 0.4416*P*] where *P* = (*F*_o_^2^ + 2*F*_c_^2^)/3
1900 reflections	(Δ/σ)_max_ < 0.001
160 parameters	Δρ_max_ = 0.25 e Å^−^^3^
0 restraints	Δρ_min_ = −0.25 e Å^−^^3^

### Special details

**Table:**

Geometry. Bond distances, angles etc. have been calculated using the rounded fractional coordinates. All su's are estimated from the variances of the (full) variance-covariance matrix. The cell esds are taken into account in the estimation of distances, angles and torsion angles
Refinement. Refinement of *F*^2^ against ALL reflections. The weighted *R*-factor *wR* and goodness of fit *S* are based on *F*^2^, conventional *R*-factors *R* are based on *F*, with *F* set to zero for negative *F*^2^. The threshold expression of *F*^2^ > σ(*F*^2^) is used only for calculating *R*-factors(gt) *etc*. and is not relevant to the choice of reflections for refinement. *R*-factors based on *F*^2^ are statistically about twice as large as those based on *F*, and *R*- factors based on ALL data will be even larger.

### Fractional atomic coordinates and isotropic or equivalent isotropic displacement parameters (Å^2^)

**Table:**

	*x*	*y*	*z*	*U*_iso_*/*U*_eq_	
Fe1	0.00000	0.00000	0.00000	0.0218 (1)	
O1	0.06876 (16)	0.10364 (9)	−0.14553 (12)	0.0289 (3)	
O2	−0.00859 (18)	0.15001 (9)	−0.35930 (13)	0.0354 (4)	
O3	−0.19925 (18)	0.08021 (10)	−0.56865 (12)	0.0349 (4)	
O4	−0.38127 (18)	−0.05047 (10)	−0.62554 (12)	0.0351 (4)	
N1	−0.51150 (18)	−0.17230 (10)	−0.08531 (13)	0.0226 (4)	
N2	−0.5674 (2)	−0.23038 (11)	0.01037 (16)	0.0345 (5)	
N3	−0.75834 (19)	−0.09910 (10)	−0.02990 (14)	0.0261 (4)	
N4	−0.16330 (18)	−0.05447 (9)	−0.17946 (13)	0.0217 (4)	
N5	−0.32936 (18)	−0.11788 (10)	−0.36005 (13)	0.0235 (4)	
C1	−0.7157 (3)	−0.18384 (14)	0.0396 (2)	0.0361 (6)	
C2	−0.6267 (2)	−0.09475 (12)	−0.10846 (16)	0.0248 (5)	
C3	−0.3469 (2)	−0.20523 (12)	−0.14359 (18)	0.0272 (5)	
C4	−0.2792 (2)	−0.12666 (11)	−0.22693 (16)	0.0216 (4)	
C5	−0.1404 (2)	0.00355 (11)	−0.28705 (16)	0.0217 (5)	
C6	−0.0165 (2)	0.09232 (12)	−0.26313 (16)	0.0245 (4)	
C7	−0.2437 (2)	−0.03540 (12)	−0.40038 (16)	0.0228 (5)	
C8	−0.2797 (2)	−0.00276 (12)	−0.54165 (17)	0.0259 (5)	
H1A	−0.78580	−0.20710	0.10290	0.0430*	
H2A	−0.61660	−0.04490	−0.17040	0.0300*	
H3A	−0.13760	0.10250	−0.50000	0.0520*	
H3B	−0.38060	−0.26360	−0.19810	0.0330*	
H3C	−0.24600	−0.22340	−0.07230	0.0330*	
H5A	−0.40260	−0.15730	−0.41090	0.0280*	

### Atomic displacement parameters (Å^2^)

**Table:**

	*U*^11^	*U*^22^	*U*^33^	*U*^12^	*U*^13^	*U*^23^
Fe1	0.0216 (2)	0.0231 (2)	0.0203 (2)	0.0023 (1)	0.0022 (1)	−0.0003 (1)
O1	0.0284 (6)	0.0284 (6)	0.0293 (6)	−0.0064 (5)	0.0031 (5)	−0.0015 (5)
O2	0.0361 (7)	0.0321 (7)	0.0375 (7)	−0.0095 (5)	0.0041 (5)	0.0108 (5)
O3	0.0412 (7)	0.0376 (7)	0.0263 (6)	−0.0011 (6)	0.0069 (5)	0.0070 (5)
O4	0.0399 (7)	0.0399 (7)	0.0240 (6)	0.0047 (6)	0.0009 (5)	−0.0041 (5)
N1	0.0236 (7)	0.0206 (7)	0.0247 (7)	0.0014 (5)	0.0070 (5)	0.0047 (5)
N2	0.0359 (8)	0.0312 (8)	0.0406 (9)	0.0099 (7)	0.0191 (7)	0.0174 (7)
N3	0.0258 (7)	0.0249 (7)	0.0282 (7)	0.0049 (5)	0.0061 (6)	0.0023 (6)
N4	0.0217 (6)	0.0217 (7)	0.0224 (6)	−0.0002 (5)	0.0053 (5)	0.0006 (5)
N5	0.0236 (7)	0.0229 (7)	0.0241 (7)	−0.0025 (5)	0.0045 (5)	−0.0039 (5)
C1	0.0365 (10)	0.0333 (10)	0.0435 (11)	0.0095 (8)	0.0214 (8)	0.0130 (8)
C2	0.0284 (8)	0.0225 (8)	0.0238 (8)	0.0040 (6)	0.0051 (6)	0.0035 (6)
C3	0.0268 (8)	0.0215 (8)	0.0360 (9)	0.0023 (6)	0.0132 (7)	0.0023 (7)
C4	0.0204 (7)	0.0205 (7)	0.0250 (8)	0.0016 (6)	0.0073 (6)	−0.0006 (6)
C5	0.0207 (8)	0.0229 (8)	0.0224 (8)	0.0007 (6)	0.0062 (6)	0.0021 (6)
C6	0.0210 (7)	0.0248 (8)	0.0289 (8)	0.0003 (6)	0.0073 (6)	0.0010 (6)
C7	0.0214 (8)	0.0248 (8)	0.0232 (8)	0.0014 (6)	0.0069 (6)	−0.0002 (6)
C8	0.0243 (8)	0.0310 (9)	0.0236 (8)	0.0079 (6)	0.0077 (7)	0.0015 (6)

### Geometric parameters (Å, °)

**Table:**

Fe1—O1	2.1452 (13)	N3—C2	1.330 (2)
Fe1—N4	2.1316 (14)	N3—C1	1.352 (2)
Fe1—N3^i^	2.2484 (15)	N4—C4	1.321 (2)
Fe1—N3^ii^	2.2484 (15)	N4—C5	1.373 (2)
Fe1—O1^iii^	2.1452 (13)	N5—C7	1.365 (2)
Fe1—N4^iii^	2.1316 (14)	N5—C4	1.345 (2)
O1—C6	1.259 (2)	N5—H5A	0.8600
O2—C6	1.255 (2)	C3—C4	1.484 (2)
O3—C8	1.308 (2)	C5—C6	1.489 (2)
O4—C8	1.213 (2)	C5—C7	1.369 (2)
O3—H3A	0.8200	C7—C8	1.480 (2)
N1—C2	1.331 (2)	C1—H1A	0.9300
N1—C3	1.471 (2)	C2—H2A	0.9300
N1—N2	1.356 (2)	C3—H3B	0.9700
N2—C1	1.310 (3)	C3—H3C	0.9700
			
O1—Fe1—N4	78.11 (6)	C7—N5—H5A	126.00
O1—Fe1—N3^i^	91.64 (6)	N2—C1—N3	114.38 (18)
O1—Fe1—N3^ii^	88.36 (6)	N1—C2—N3	109.91 (14)
O1—Fe1—O1^iii^	180.00	N1—C3—C4	111.65 (13)
O1—Fe1—N4^iii^	101.89 (6)	N5—C4—C3	125.00 (14)
N3^i^—Fe1—N4	90.69 (6)	N4—C4—N5	110.69 (14)
N3^ii^—Fe1—N4	89.31 (6)	N4—C4—C3	124.31 (15)
O1^iii^—Fe1—N4	101.89 (6)	N4—C5—C7	109.26 (14)
N4—Fe1—N4^iii^	180.00	N4—C5—C6	118.24 (14)
N3^i^—Fe1—N3^ii^	180.00	C6—C5—C7	132.51 (15)
O1^iii^—Fe1—N3^i^	88.36 (6)	O1—C6—O2	125.73 (15)
N3^i^—Fe1—N4^iii^	89.31 (6)	O1—C6—C5	116.10 (14)
O1^iii^—Fe1—N3^ii^	91.64 (6)	O2—C6—C5	118.15 (15)
N3^ii^—Fe1—N4^iii^	90.69 (6)	C5—C7—C8	133.23 (15)
O1^iii^—Fe1—N4^iii^	78.11 (6)	N5—C7—C5	105.78 (14)
Fe1—O1—C6	116.15 (11)	N5—C7—C8	120.92 (14)
C8—O3—H3A	109.00	O3—C8—O4	122.94 (16)
N2—N1—C3	117.39 (13)	O3—C8—C7	116.19 (14)
C2—N1—C3	133.07 (14)	O4—C8—C7	120.86 (15)
N2—N1—C2	109.52 (13)	N2—C1—H1A	123.00
N1—N2—C1	103.17 (15)	N3—C1—H1A	123.00
Fe1^iv^—N3—C1	123.27 (13)	N1—C2—H2A	125.00
Fe1^iv^—N3—C2	133.72 (11)	N3—C2—H2A	125.00
C1—N3—C2	103.01 (15)	N1—C3—H3B	109.00
Fe1—N4—C5	111.20 (10)	N1—C3—H3C	109.00
C4—N4—C5	106.21 (13)	C4—C3—H3B	109.00
Fe1—N4—C4	142.58 (11)	C4—C3—H3C	109.00
C4—N5—C7	108.07 (13)	H3B—C3—H3C	108.00
C4—N5—H5A	126.00		
			
N4—Fe1—O1—C6	3.33 (11)	Fe1—N4—C4—C3	3.5 (3)
N3^i^—Fe1—O1—C6	93.69 (12)	C5—N4—C4—N5	0.72 (17)
N3^ii^—Fe1—O1—C6	−86.31 (12)	C5—N4—C4—C3	−178.19 (14)
N4^iii^—Fe1—O1—C6	−176.67 (11)	Fe1—N4—C5—C6	−1.63 (17)
O1—Fe1—N4—C4	177.56 (18)	Fe1—N4—C5—C7	178.49 (10)
O1—Fe1—N4—C5	−0.67 (10)	C4—N4—C5—C6	179.49 (13)
N3^i^—Fe1—N4—C4	86.04 (18)	C4—N4—C5—C7	−0.39 (17)
N3^i^—Fe1—N4—C5	−92.20 (11)	C7—N5—C4—N4	−0.78 (18)
N3^ii^—Fe1—N4—C4	−93.96 (18)	C7—N5—C4—C3	178.12 (14)
N3^ii^—Fe1—N4—C5	87.80 (11)	C4—N5—C7—C5	0.50 (17)
O1^iii^—Fe1—N4—C4	−2.44 (18)	C4—N5—C7—C8	−176.74 (14)
O1^iii^—Fe1—N4—C5	179.33 (10)	N1—C3—C4—N4	84.25 (18)
Fe1—O1—C6—O2	173.29 (13)	N1—C3—C4—N5	−94.50 (18)
Fe1—O1—C6—C5	−5.11 (17)	N4—C5—C6—O1	4.6 (2)
C2—N1—N2—C1	0.14 (19)	N4—C5—C6—O2	−173.91 (14)
C3—N1—N2—C1	−178.66 (15)	C7—C5—C6—O1	−175.54 (16)
N2—N1—C2—N3	0.34 (18)	C7—C5—C6—O2	5.9 (3)
C3—N1—C2—N3	178.87 (16)	N4—C5—C7—N5	−0.07 (17)
N2—N1—C3—C4	−169.17 (14)	N4—C5—C7—C8	176.68 (16)
C2—N1—C3—C4	12.4 (2)	C6—C5—C7—N5	−179.92 (15)
N1—N2—C1—N3	−0.6 (2)	C6—C5—C7—C8	−3.2 (3)
C2—N3—C1—N2	0.8 (2)	N5—C7—C8—O3	176.25 (14)
Fe1^iv^—N3—C1—N2	−178.52 (12)	N5—C7—C8—O4	−2.5 (2)
C1—N3—C2—N1	−0.64 (18)	C5—C7—C8—O3	−0.1 (3)
Fe1^iv^—N3—C2—N1	178.54 (11)	C5—C7—C8—O4	−178.83 (17)
Fe1—N4—C4—N5	−177.57 (13)		

Symmetry codes: (i) *x*+1, *y*, *z*; (ii) −*x*−1, −*y*, −*z*; (iii) −*x*, −*y*, −*z*; (iv) *x*−1, *y*, *z*.

### Hydrogen-bond geometry (Å, °)

**Table:**

*D*—H···*A*	*D*—H	H···*A*	*D*···*A*	*D*—H···*A*
O3—H3A···O2	0.82	1.70	2.5175 (19)	179
N5—H5A···N2^v^	0.86	2.01	2.850 (2)	166

Symmetry codes: (v) *x*, −*y*−1/2, *z*−1/2.
